# Introduction to the article collection ‘Translation in healthcare: ethical, legal, and social implications’

**DOI:** 10.1186/s12910-016-0157-6

**Published:** 2016-11-14

**Authors:** Michael Morrison, Donna Dickenson, Sandra Soo-Jin Lee

**Affiliations:** 1 Centre for Health, Law and Emerging Technologies (HeLEX), Nuffield Department of Population Health, University of Oxford, Ewert House Banbury Road, Oxford, OX2 7DD UK; 2 Centre for Medical Ethics, University of Bristol, Bristol, UK; 3 Center for Biomedical Ethics, Stanford University School of Medicine, 1215 Welch Road MOD A Office 75, Stanford, CA 94305 USA

## Abstract

New technologies are transforming and reconfiguring the boundaries between patients, research participants and consumers, between research and clinical practice, and between public and private domains. From personalised medicine to big data and social media, these platforms facilitate new kinds of interactions, challenge longstanding understandings of privacy and consent, and raise fundamental questions about how the translational patient pathway should be organised.

This editorial introduces the cross-journal article collection "Translation in healthcare: ethical, legal, and social implications", briefly outlining the genesis of the collection in the 2015 Translation in healthcare conference in Oxford, UK and providing an introduction to the contemporary ethical challenges of translational research in biology and medicine accompanied by a summary of the papers included in this collection.

## Challenges and opportunities: introducing translation in healthcare

In June 2015 some 130 delegates from 20 countries including the USA, Japan, Taiwan, Israel, and Canada, converged on Oxford for the *Translation in Healthcare* conference, hosted by the Centre for Health, Law and Emerging Technologies (HeLEX) at the University of Oxford. The conference, subtitled ‘*exploring the impact of emerging technologies*’, provided a forum for a range of voices from different national and disciplinary perspectives to discuss the ethical, legal and social challenges raised by novel healthcare technologies. Hosted in the spacious, contemporary environs of the recently-opened Andrew Wiles building, the event deployed a number of innovative features, including works by artist in residence Miranda Creswell and an interactive *Écouter* session for conference attendees (about which more below). The intention was to foster the kinds of lively, productive discussions that are otherwise often restricted to the intervals between scheduled presentations. The conference was also part of the international ELSI 2.0 collaboratory [1] with webcasts of key plenaries and a live Twitter feed connecting the debates with a global audience. Some of the papers in this special issue were presented in an early form at the *Translation in Healthcare* conference, while others germinated from the interdisciplinary exchanges and discussions stimulated by the event. Each paper in this special issue addresses a particular technology and brings a particular disciplinary and methodological approach, but together they reveal the broad array of ethical issues emerging from the work of translation.

In recent years the notion of ‘translation’ has been ever-present in discussions about healthcare technology and biomedical research, becoming something of a mantra for policymakers and funders to rank alongside ‘innovation’ and, indeed, ‘choice’. As with other concepts that have achieved near-ubiquity, ‘translation’ appears to mean different things to different people in different contexts [2, 3]. However, it is most commonly understood to refer to the application of novel scientific discoveries to improve health outcomes, benefit patients, produce new products and services, and promote economic growth and prosperity. This meaning of translation is reflected in the oft-stated goal of helping innovative discoveries move ‘from bench to bedside’. The translational ideal encompasses both a hopeful optimism about the potential of technology to transform health and alleviate suffering and a concern that the benefits of science are in danger of failing to be realised. These hopes and fears for the future are employed to stimulate and organise action in the present [4]. This mixture of hope and concern as a stimulus for widespread change in the organisation of research is clearly illustrated by the field of genomics.

On the one hand, the sequencing of the complete human genome has been hailed as a landmark scientific achievement with the potential to “revolutionise the diagnosis, prevention and treatment of most, if not all, human diseases” [5]. On the other, some have questioned whether those anticipated benefits have so far been adequately demonstrated, especially in terms of returns for patients with chronic and incurable conditions [6]. When, in 2003 the US National Institutes of Health (NIH) launched its ‘roadmap’ for future research, the sequencing of the human genome was presented as both an *opportunity* and a *challenge*; realizing the benefits of this and other scientific discoveries would not happen automatically, but required work, effort and transformation of existing systems of clinical research to drive the translation of discoveries into clinical benefits [7]. In fact, nothing less than the ‘reengineering of the clinical enterprise’ was prescribed ([7], p64). The changes, which have resulted from the roadmap and from similar endeavours elsewhere, in effect constitute the translational enterprise.

## From networked biobanks to citizen science: the changing landscape of biomedical research

These changes have been, and continue to be, substantial and extend to many domains of health-related science and technology beyond genomics. Many of the initiatives put in place to support translational research have been ‘top down’ changes fostered by state governments and major organisations with significant financial and institutional resources. In the USA the NIH Clinical and Translational Science Awards (CTSA) Program has established 50 regional hubs to support translational research and had a budget in 2016 of USD 685 million to provide training, engage under-served populations, and develop bioinformatics and other tools to improve translational efficiency. The Innovative Medicines Initiative is a joint venture between the European Commission and the European Federation of Pharmaceutical Industries and Associations (Efpia) with a Euro 3.3 billion budget to support public- private consortia working on novel health technologies and platforms for drug discovery.

Translational research also requires large data sets and large cohorts of research participants. In the UK, *Genomics England* has been set up to sequence the genomes of 100,000 volunteers. China’s *Kadoorie Biobank* already has half a million registered participants, while in the USA President Obama’s *Precision Medicine Initiative* has started recruiting the first of an envisioned 1 million patients and healthy volunteers who will contribute biological, environmental, lifestyle and other data. It is not only states that are instituting large-scale collections of health and biomedical data; *AstraZeneca* recently announced a collaborative venture with the *Wellcome Trust Sanger Institute* and Craig Venter’s San Diego-based biotech *Human Longevity* to collate genome sequence data and health records from 2 million people to help identify rare genetic variations with significant effects for health [8]. In addition to generating large swathes of new data, translational efforts have also focused on improving access to existing data.

Much of the impetus behind the open access and open data movements is driven by a belief that increased availability of scientific data will lead to new knowledge and new applications [9]. New infrastructures and networks have also been developed to facilitate the sharing of scientific data and expertise such as the *Biobanking and Biomolecular resources Research Infrastructure* (BBMRI-ERIC) for international biobanking ([10], the *Human Heredity and Health in Africa* (H3Africa) network and the *Global Alliance for Genomics and Health* (GA4GH) for genomic and clinical data [11]. Another example is the increasing use of large consortia as an organisational form of science intended to foster collaboration between disciplines, and between industrial and academic researchers, to tackle major translational tasks [12, 13].

Considered broadly, the transformations brought about by the desire to accelerate the translation of research into new treatments also have a ‘bottom up’ component, driven by the efforts of patients and citizens motivated to secure improvements in healthcare. One element of this participatory turn in translation is reflected in the rise of novel digital platforms where patients can share experiences and data, including genomic data, on their conditions, such as *PatientsLikeMe* and the *Platform for Engaging Everyone Responsibly* (PEER) developed by the Genetic Alliance patient group. Many common translational aims are also shared by advocates of Citizen Science movements, such as the *Mark2Cure* initiative which teaches lay members to scan and interpret biomedical literature, or do-it-yourself biology and bioinformatics entrants in the *International Genetically Engineered Machine* (IGEM) competition, even if they do not necessarily use the term ‘translational research’. The power of distributed data contributed by patients and citizens chimes with the demand for large data sets in translational research, and the drive to engage wider publics in data generation efforts has attracted interest from academic, government, and commercial organisations alike. This is evident in the rise of patient-centred initiatives in biomedical research [14], but also in the business models of companies like *23andMe*, which employed a model of active customer participation in data production in order to generate their proprietary databases of genomic and lifestyle information [15, 16]. Indeed, the push for greater engagement and participation is forging novel and unexpected alliances and forms of interaction such as the *European Patients’ Academy on Therapeutic Innovation* (EUPATI) a combined venture of European patient groups, universities and pharmaceutical companies, funded through the IMI program [17].

## Ethical, legal and social issues in translation

These translational transformations are reconfiguring traditional boundaries: between patient, research participant and consumer; lay people and experts; medical research and clinical practice; and between the public and private domains. New configurations of technologies, service providers and users challenge existing regulatory categories, present novel opportunities and risks, and raise important ethical questions. Increased sharing of personal medical and biological information and increasingly international movements of data raise issues of privacy and security, but also challenge the adequacy of traditional ethical concepts like consent, and, indeed, justice. Many large scale data gathering operations rely on a broad consent to use and reuse data for multiple purposes [18, 19]. This concept challenges established understandings of what informed consent is intended to mean, and raises the possibility that the protections it is supposed to offer may be undermined. Similarly, global, networked flows of data are also redefining the meaning of other traditional protections of human subjects’ research such as the right to withdraw from participation [20]. What are the implications of these changes for public trust and accountability in research? What governance options are afforded, and which capabilities are required, by the digital and algorithmic processing of data on a global scale? And is there a danger that the increasing focus on individual biological and lifestyle causes of disease might overshadow efforts to address environmental and systemic determinants of illness [21]?

As translational endeavours foster new kinds of engagement between doctors, scientists, patients, citizens, states and companies it is important to consider how this affects what it means to be engaged in research. Does translational research carry new normative requirements? Is there a moral duty to participate, to give up and give away personal information for a greater good? What ethical challenges are involved as public and patient engagement processes are applied outside European and North American contexts? And what do these changes mean for scientists, regulators, research ethics committee/institutional review board members, healthcare professionals and academics? It is particularly relevant to consider the impact of these changes on populations who are already marginalised or under-represented in medical research [22, 23]. This applies not only to different communities within developed countries, but to the wider flow of data, materials, technology and medical knowledge between the global North and South [24, 25]. Do translational initiatives risk exacerbating existing inequalities and what means of redress might be available? Moreover, are some forms of contribution, such as participation in data sharing networks, welcomed while other approaches such as DIY Biology are regarded with suspicion or even hostility [26]?

The papers in this thematic collection take up the challenge of developing an ethical analysis of translational research. The importance of genomics as a key site of translational endeavours is reflected in the organisation of this thematic collection, which is shared across *BMC Medical Ethics* and *BMC Medical Genomics*. Several articles focus on the emerging challenges of biobanking and big data through the linkage of bio-specimens and medical and non-medical data. Providing a comparative international perspective, Chalmers et al. offer a review of biobanking governance through the lens of the diverse regulatory contexts of seven countries. The authors chart four waves of biobanking management and policy that have resulted in response to the challenges of ensuring informed consent, standardization, sustainability and public trust [27]. Addressing similar concerns by drawing attention to the lack of regulatory oversight of unauthorized secondary uses of health data and samples, O’Doherty et al. argue that without building ethical oversight in tandem with the proliferation of health data collections intended for research, unintended negative consequences are likely to result [28].

Careful consideration of the ethical implications of big data for clinical decision-making is addressed by Fischer et al., who identify the epistemological questions that ‘systems medicine’ and the use of bioinformatics tools and algorithms raise for patient care [29]. This theme of unintended consequences is taken up in by Newson et al. in their call for greater engagement with the normative questions of ambiguous genomic information. Using examples of clinical case scenarios, the authors argue for a reframing of uncertain test results and propose that healthcare providers directly engage clinical ambiguity as inherent to genomic medicine [30]. There is also a potential conflict between data-sharing and the concern to prevent data being used to promote bioterrorism, as Bezuidenhout and Morrison argue [31]. ‘Dual use’ policies address the latter need, but they are rarely discussed in comparison with the ideal of open access, as this paper does.

Several authors tackle the theme of participation in translational research. Greater public and patient involvement in research, as providers of personal genetic, health and lifestyle information, is deeply embedded in many models of precision or personalised medicine. However, as Nicholls and colleagues note, there are also compelling normative, political and practical reasons to engage with various publics about how novel medical technologies and services should be delivered. Blasimme and Vayena develop the conceptual aspects of this idea, exploring the implications of precision medicine initiatives for current understandings of autonomy, and arguing that greater participation may also require offering more meaningful choices to participants in medical research [32]. New modes of engagement are also in evidence; Murtagh et al. describe the results of an *Employing COnceptual schema for policy and Translational Engagement in Research* (Écouter) session which was run with the participants during the Translation in Healthcare conference itself [33]. The Écouter model involves a digital mind-mapping process in which participants offer their own responses to an initial question and a set of ‘seeded’ prompts in the form of texts, images, videos and so forth. The system is designed to be adaptable to face to face or entirely online settings. The latter are especially pertinent given that so many translational endeavours from genomic data-sharing to wearable smart devices involve digital data collection and dissemination.

Early-career researchers in the ELSI field face particular problems of isolation from disciplinary support structures, as the article by Bell et al. reports [34], based on a workshop for ELSI researchers at the Translation in Medicine conference. This workshop discussed the potential opportunity to use web 2.0 technologies, such as the ELSI 2.0 workspace currently provided through the Global Health network [35], to transform academic support structures and address some of the challenges faced by ELSI ECRs, by helping to facilitate mentoring and support, access to resources and new accreditation metrics. At the same time, it is worth remembering that a mandate for digital engagement cannot always be taken for granted. The study by Coathup and colleagues describes the findings of a survey carried out with Myotonic Dystrophy patients in Japan to gauge the acceptability of using digital methods to foster greater communication with healthcare professionals [36]. A majority of study participants reported an interest in receiving more up-to-date information about the latest medical developments relating to their condition, but also wanted to ensure the confidentiality and security of their communications and to be able to remain in control of the interactive process.

The remaining papers in the collection explore the views, attitudes and concerns of different groups involved in the translational process. Bertier, Hétu and Joly [37] review the literature on whole exome sequencing to identify the key concerns for clinicians and researchers. Woolley et al. [38] critically assess the frequently invoked rhetoric of ‘citizen science’ and ‘participant-led research’, comparing US and UK case studies and analysing the role of commercial companies in encouraging a participatory strategy. Budin-Ljøsne and Harris report on European Patient Interest Organisations’ perceptions of personalised medicine [39], and Nicholls et al. discuss how Canadians regarded the prospect of incorporating genomic risk predictions for cancer and childhood diabetes into routine clinical practice. [40] These studies illustrate how hopes and concerns vary considerably across disease groups, technologies, and the envisioned roles of end users (patients, clinicians, and those mediating between groups). While this highlights the need for inclusive, deliberative models of governance that incorporate the needs of different stakeholders in the translational process, these studies also show that there are some common areas of concern (albeit often expressed in different terms) such as the likely cost of new personalised treatments and how this might affect access.

Taken together, the papers in this thematic collection represent a concerted attempt to open up the ideas, practices and technologies of biomedical translation to ethical scrutiny. We do not claim that this is a comprehensive analysis; a range of aspects of translation have been identified and evaluated, but many other facets await further consideration. If this collection has a further insight to offer beyond the theoretical and empirical work presented in individual papers, it is, hopefully, to illustrate the value of recognising translation as an ethically significant phenomenon in itself, as something that transcends particular fields, cases and issues even as it transforms them, and that has wide-ranging implications for the responsible practice of the life sciences.

A small number of additional papers remain under review for this thematic collection and may be appended to the online Translation in healthcare page in future.
